# Genetic polymorphisms of patients on stable warfarin maintenance therapy in a Ghanaian population

**DOI:** 10.1186/s13104-016-2306-x

**Published:** 2016-12-09

**Authors:** William Kudzi, Samuel Yao Ahorhorlu, Bartholomew Dzudzor, Edeghonghon Olayemi, Edmund Tetteh Nartey, Richard Harry Asmah

**Affiliations:** 1 Centre for Tropical Clinical Pharmacology and Therapeutics, School of Medicine and Dentistry, College of Health Sciences, University of Ghana, P.O. GP 4236, Accra, Ghana; 2 Department of Medical Biochemistry, School of Biomedical and Allied Health Sciences, College of Health Sciences, University of Ghana, P.O. GP 143, Accra, Ghana; 3 Department of Haematology, School of Medicine and Dentistry, College of Health Sciences, University of Ghana, P.O. GP 4236, Accra, Ghana; 4 Department of Medical Laboratory Sciences, School of Biomedical and Allied Health Sciences, College of Health Sciences, University of Ghana, P.O. GP 143, Accra, Ghana

**Keywords:** Warfarin, Allele frequencies, *CYP2C9*, *CYP4F2*, *VKORC1*, Pharmacogenetics

## Abstract

**Background:**

Warfarin is a widely prescribed anticoagulant with narrow therapeutic window for thromboembolic events. Warfarin displays large individual variability in dose requirements. The purpose of this study is to assess the contribution of patient-specific and genetic risk factors to dose requirements of patients on either high or low warfarin maintenance dose in Ghana. Blood samples were collected from 141 (62 males, 79 females) Ghanaian patients on stable warfarin dose to determine their INR. Influence of patient specific factors and gene variations within *VKORC1*, *CYP2C9* and *CYP4F2* were determined in patients on either high or low warfarin maintenance dose.

**Results:**

One hundred and forty-one patients took part in the study with 79 (56%) participants being Female. The median age of the study participants was 48 years [IQR: 34–58]. The median duration for patients to be on warfarin therapy was 24 months [IQR: 10–72]. Majority of the study participants (80.9%, n = 114) did not have any side effects to warfarin. CYP2C9*2 and CYP2C9*3 variant alleles were not detected. *VKORC1* variant allele was observed at 6% and *CYP4F2* variant allele was observed at 41%. Duration of patients on warfarin therapy was marginally associated with high warfarin dose (adjusted OR = 1.01 [95% CI 1.00–1.02], p = 0.033) while the odds of heterozygous individuals (G/A) for VKORC1 gene to have high warfarin dose compared to persons with homozygous (G/G) (adjusted OR = 0.06 [95% CI 0.01–0.63], p = 0.019). Age, gender, diagnosis, presence of side effects and other medications were not associated with warfarin dose (p = 0.05).

**Conclusion:**

This study provides data on *VKORC1* and *CYP4F2* variants among an indigenous African population. Duration of patients on warfarin therapy was marginally associated with high warfarin dose. CYP2C9*2 and *3 variants were not detected and may not be the most important genetic factor for warfarin maintenance dose among Ghanaians.

## Background

Warfarin is an oral anticoagulant agent used worldwide for preventing and managing thromboembolic events that often give rise to stroke, deep vein thrombosis (DVT) and pulmonary embolism (PE). It is also used following heart valve replacements and atrial fibrillation (AF) [1]. The use of Warfarin has increased over the last 15 years, especially among the elderly population [2]. Warfarin has a narrow therapeutic range which varies among individual patients making selection of the right warfarin dose at the onset of treatment challenging. An appropriate warfarin dose in one patient may induce a haemorrhagic event in another. Clinical factors, demographic variables, and variations in genes contribute significantly to the variable warfarin dose requirements among patients [3].

It is estimated that 1% of patients die due to bleeding complications associated with warfarin and up to 15% of patients experience minor bleeding complications [4]. The efficacy of warfarin is dependent on maintaining a patient’s anticoagulation within acceptable therapeutic range without the risk of bleeding. The effectiveness and safety of warfarin is dependent on maintaining the international normalized ratio (INR), within the recommended therapeutic range of 2.0 and 3.0 for most conditions and 2.5 and 3.5 following heart valve replacements [5].

This large variation in warfarin dose requirements among patients may be due to concomitant medications, gender, nutritional status, alcohol consumption, liver disease, hyperthyroidism, Congestive heart failure and variations in genes [3].

Several studies have indicated that elderly patients require lower warfarin doses compared to younger ones [6] while women require lower warfarin dose [7]. Recent studies have also suggested that populations from the African American require more warfarin to maintain their INR between 2 and 3 than do Caucasians [5, 8].

Pharmacogenomics research on warfarin has focused on vitamin K epoxide reductase (*VKORC*), Cytochrome P450 isozyme 2C9 (*CYP2C9*), and Cytochrome P450 isozyme 4F2 (*CYP4F2*) genes [9]. Warfarin is a racemic mixture of R- and S-enantiomers. The S-warfarin enantiomer is more potent and is metabolized in the liver by CYP2C9 enzyme [10]. CYP2C9*2 (430C > T and rs 1,799,853) and CYP2C9*3 (1075A > C and rs 1,057,910) are common variants known to decrease warfarin maintenance dose requirement in patients. CYP2C9*2 and CYP2C9*3 variant alleles have been reported at 3.3 and 2.3% respectively in African American populations [11]. CYP2C9*2 variant allele has rarely been reported in Asian populations while the CYP2C9*3 variant allele was prevalent at 1.1–6.8% within the Asian populations [12]. Patients carrying CYP2C9*2 and CYP2C9*3 alleles potentially have a greater risk of bleeding during initiation of warfarin and subsequently require lower doses [13]. Many studies have shown the use of *CYP2C9* polymorphism as being helpful in optimizing the administration of warfarin [13, 14].

Cytochrome P450-4F2 (*CYP4F2*) gene also contributes 1–2% of warfarin dose variability and impact on stable warfarin dose [15]. Patients with two variant TT alleles of *CYP4F2,* (p. V433M and rs 2,189,784), will require approximately 1 mg/day more of warfarin than those who carry two wild type CC alleles [16].

The anticoagulant activity of warfarin is due to inhibition of the vitamin K epoxide reductase complex subunit 1 (*VKORC1*) enzyme which reduces the regeneration of vitamin K and thus exerting its anticoagulation effect. Polymorphism of g.-1639G > A (rs 9923231) within *VKORC1* promoter reduces the expression of the gene and therefore lowers the amount of VKORC and leads to warfarin sensitivity. Variations in *VKORC1* have been associated with both warfarin sensitivity and warfarin resistance. The prevalence of *VKORC1* polymorphism has been reported in literature as 37% for Caucasians and 14% for Africans [8]. *CYP2C9* and *VKORC1* polymorphisms occur frequently in patients who are warfarin “sensitive” and require lower doses, whereas patients with *VKORC1* missense mutations are warfarin “resistant” and require higher doses [17]. CYP2C9*2, CYP2C9*3 and *VKORC1* promoter mutation together is estimated to account for 40–63% of the variability in therapeutic warfarin dose [8, 18]. Combination of genetic and other clinical factors to predict the warfarin maintenance dose may be more accurate than using clinical factors alone [19].

Clinical testing of drugs and majority of clinical research are performed in Europe and the USA where individuals of African descent are in minority and the populations are underrepresented in these research activities [20]. Paying attention to pharmacogenomics research in Sub-Saharan African population is particularly important because of the increasing numbers of communicable diseases such as HIV/AIDS and non-communicable diseases such as hypertension. Pharmacogenetic data among the indigenous African population is scarce and there is currently no pharmacogenetic data on warfarin metabolism among the Ghanaian population. Allele frequencies among different ethnic patient populations vary and are unclear. Frequencies of *CYP4F2, CYP2C9* and *VKORC1* among indigenous African populations have not been systematically established. This study seeks to determine how various patient specific factors impact management of warfarin dose in Korle-bu Teaching Hospital. The study also sought to determine and compare the impact of polymorphisms of *CYP2C9*, *VKORC1* and *CYP4F2 on* patients on either low or high warfarin maintenance dose in Ghana.

## Methods

### Subjects

The study population comprised of 141 (62 males, 79 females) stable warfarin patients recruited mainly from the Cardiothoracic Center of the Korle-bu Teaching (KBTH) in Accra, Ghana. The Cardiothoracic Center screens about 220 patients a week and all adults patients (18 years and above) who were on warfarin therapy were eligible for recruitment into the study. These eligible patients were screened by a Clinician and only warfarin patients whose warfarin dose requirement remained stable for at least 3 previous clinic visits over a minimum period of 3 months, with INR within the range of 2.0–3.0, were included in the study. Warfarin patients with unstable dose requirement, with target INR outside the range, were deem as noncompliant to warfarin therapy and were excluded from the study. Ethical and Protocol Review Committee of School of Medicine and Dentistry, University of Ghana, approved this study with reference number MS-Et/M.6-P.4.5/2011-2012. Written informed consent was obtained from all patients prior to inclusion in the study.

Patients between 17 and 77 years of age receiving warfarin and attending clinics for permanent Atrial Fibrillation/Flutter, Left atrial or ventricular thrombus, Deep Vein Thrombosis, Pulmonary Embolism, Heart Valve Replacement (Mechanical or Biological with AF), Cardiomyopathy (Ischemic or Dilated), and Peripheral Vascular Disease were enrolled for the study. Patients with the following medical conditions; history of gastro-intestinal (GI) bleeding or peptic ulcer disease, significant liver disease (active hepatitis or chronic hepatitis B or C virus (HBV/HCV) infection), uncontrolled hypertension, chronic diarrhoea or malabsorption syndrome, viral or bacterial infection prior to enrolment, active or previous infective endocarditis, hospital stay >30 days as a result of septicaemia, mediastinitis or pneumonia, cardiac cachexia, and morbid obesity were excluded from the study.

Patient information such as age, gender, clinical history for warfarin dose, present INR, additional medical problems and all other medications were collected retrospectively from chart reviews. Height and weight were measured to calculate body mass index (BMI) for all patients.

Patients were classified as being on either a high or low depending on their daily warfarin maintenance doses to determine areas where the two populations differed [21]. High daily warfarin dose was defined as >5 mg/daily and a low daily warfarin dose was defined as ≤5 mg/daily based on common practice already available in Korle-Bu Teaching Hospital.

Eighty-four (84) of these patients were on low daily warfarin maintenance dose and fifty-seven (57) of these patients were on high daily warfarin maintenance dose. Blood sample (3 ml) was taken from all participating patients for INR measurement and for *CYP2C9*, *VKORC1* and *CYP4F2* genotyping.

### DNA extraction and genotyping genomic

DNA was isolated from 1.5 ml of whole blood sample collected in tubes containing ethylenediaminetetraacetic acid (EDTA) and stored at 4 °C using a QIAamp DNA blood Maxi Kit (Qiagen, Crawley UK), following the manufacturer’s protocol. CYP2C9*2 and CYP2C9*3 variant alleles were analysed using Polymerase Chain Reaction–Restriction Fragment Length Polymorphism (PCR–RFLP) as previously described by Burian et al. [22] with some modifications. *CYP2C9* allele (*1) was assigned as the wild-type in the absence of other detectable variant alleles. The presence of *VKORC1* (rs 9,923,231) variant allele was determined by PCR–RFLP as previously described by Aomori et al. [23] and *CYP4F2* (rs 2,108,622) variant allele determined as described by Cen et al. [9].

All PCRs were carried out in 25 µl final volume containing 0.5 g genomic DNA, 0.025 µM forward and reverse primers and 12.5 µl of 2× Taq Super mix (400 µM dNTPs, 1.5 mM Mg^2+^, 1 U Taq polymerase) (BioPioneer, USA). Details of primer sequences, amplicon sizes and restriction enzymes for *CYP2C9*, *CYP4F 2* and *VKORC1* are shown in Table 1. PCR cycling condition for CYP2C9*2 allele consisted of initial denaturation step of 95 °C for 10 min, followed by 45 cycles (95 °C for 5 s, 53 °C for 10 s, 72 °C for 15 s) and final extension at 72 °C for 5 min. Cycling condition for CYP2C9*3 allele carried out at an initial denaturation step of 5 min at 94 °C, followed by 30 cycles (94 °C for 45 s, 53 °C for 45 s, and 72 °C for 1 min) with a final extension for 5 min at 72 °C. The amplifications for *CYP4F2* (rs 2,108,622) consisted of an initial denaturation step at 95 °C for 5 min followed by 35 amplification cycles (94 °C for 30 s, 50 °C for 30 s and 72 °C for 1 min) and a final incubation at 72 °C for 7 min. The amplifications for *VKORC1*, initial denaturation step of 5 min at 95 °C, followed by 35 cycles (95 °C for 1 min, 51 °C for 30 s, and 72 °C for 2 s) with a final extension for 10 min at 72 °C. Blank tubes without DNA were included in each batch of samples analysed as control.

**Table 1:** Primer sequences

	Sequences	Amplicon size	Restriction enzyme	Reference
CYP2C9*2				
Forward	5′-CACTGGCTGAAAGAGCTAACAGAG-3′	375 bp	*Ava*II	[22]
Reverse	5′-GTGATATGGAGTAGGGTCACCCAC-3′			
CYP2C9*3				
Forward	5′-TGCACGAGGTCCAGAGGTAC-3′	105 bp	*Kpn*I	[22]
Reverse	5′-ACAAACTTACCTTGGGAATGAGA-3′			
*CYP4F2*				
Forward	5′-CGGAACTTGGACCATCTACA-3′	439 bp	*Pvu*II	[14]
Reverse	5′-CCTACTCTCCCACAGGCATTA-3′			
*VKORC1*				
Forward	5′-ATCCCTCTGGGAAGTCAAGC-3′	636 bp	*Nci*I	[23]
Reverse	5′-CACCTTCAACCTCTCCATCC-3′			

The resulting PCR products were digested with appropriate restriction enzymes (Table 1). All digested products were visualized on 2.5% agarose gels stained with ethidium bromide. A few randomly selected samples of genotypes were sequenced for confirmation of assays after restriction digests.

### Statistical analysis

All data were entered into Statistical Package for Social Science (ver.17.0; SPSS, Chicago, IL) and imported into Stata™ version 10 (StataCorp, College Station, Texas, United States) for statistical analyses. Descriptive statistics were calculated for patients on both high and low warfarin dose. Data were summarized as frequencies and proportions. Genotype deviations from the Hardy–Weinberg equilibrium were determined. Chi-square tests were performed to test for association between categorical variables. Warfarin dosages were summarized as means with accompanying standard deviations, and compared between patients of different genotypes using t tests and analysis of variance (ANOVA). All reported p values were two-sided and considered statistically significant at a level of p < 0.05.

## Results and discussions

### Patient characteristics

Table 2 summarizes the baseline socio-demographics and clinical characteristics of the 141 patients who consented to take part in the study. The median age of the study participants was 48 years [IQR: 34–58]. Female participants were 79 (56%) and the most common indications for warfarin use were valve replacement (n = 63, 45%), deep vein thrombosis (n = 51, 36.4%), pulmonary embolism (n = 16, 11.4%), and atrial fibrillation (n = 10, 7.20%). The median duration for patients to be on warfarin therapy was 24 months [IQR: 10–72]. Majority of the study participants (80.9%, n = 114) did not have any adverse reaction to warfarin.

**Table 2:** Socio-demographic and clinical characteristics of 141 patients administered warfarin at Korle-bu Teaching Hospital in Accra

Characteristic	Frequency, %
Age (years) (N = 141)	
Median (inter-quartile range)	48 (34–58)
Gender (N = 141)	
Female	79 (56.0)
Body mass index (N = 141)	
Normal (18.00–24.99 kg/m^2^)	55 (39.0)
Overweight/Obese (≥ 25.00 kg/m^2^)	86 (61.0)
Warfarin duration (months) (N = 139)	
Median (inter-quartile range)	24 (10–72)
Diagnosis (N = 140)	
Mitral valve replacement	63 (45.0)
Deep vein thrombosis	51 (36.4)
Atrial fibrillation	10 (7.2)
Pulmonary embolism	16 (11.4)
Presence of side effects (N = 141)	
Yes	27 (19.1)
No	114 (80.9)
Other medication given (N = 141)	
Yes	76 (53.9)
No	65 (46.1)

*N* number of study participants

Allele and genotype frequencies for *CYP2C9*, *CYP4F2* and *VKORC1* variants genotyped are summarized in Table 3. A total of 141 samples were collected for analysis, however, between 87 and 116 were available for each single nucleotide polymorphism. All the genotypes were in Hardy–Weinberg equilibrium. CYP2C9*2, and CYP2C9*3 variant alleles were not detected in any of the patients genotyped in this study population. This is consistent with data from an earlier study among Ghanaians [24] and Beninese population [25]. Frequencies of *VKORC1* allele A was observed at 6.2% which is similar to that reported for Mozambicans (3.5%) [26] and African–Americans (10.8%) [27]. It is however lower than that reported for Asians (66.7%), Caucasians (40.6%), Hispanics (43.6%) [17] and Ashkenazi Jewish (46.7%) populations [28]. CYP2C9*5, *6, *8, *11 variants occur individually in relatively low allele frequencies among African populations and have been associated with warfarin dose. These variants were not analyzed in this study, homozygous CYP2C9*1 or heterozygous CYP2C9*1 were therefore considered putative. An earlier study among Ghanaian population did not detect CYP2C9*5 but reported CYP2C9*11 with the frequency of 2% [24].

**Table 3:** Allele and Genotype frequencies (VKORC1, CYP4F2, CYP2C9*2 and CYP2C9*3) of patients administered warfarin at Korle-bu Teaching Hospital in Accra

Genetic characteristic	Frequency, %
*VKORC1* (N = 96)	
Gene	
GG (wild type)	84 (87.5)
GA	12 (12.5)
AA	–
Allele	
G	180 (93.8)
A	12 (6.2)
*CYP4F2* (N = 116)	
Gene	
CC (wild type)	28 (24.1)
CT	80 (69.0)
TT	8 (6.9)
Allele	
C	136 (58.6)
T	96 (41.4)
CYP2C9*2 (N = 86)	
Gene	
CC (wild type)	86 (100)
CT	–
TT	–
Allele	
C	172 (100)
T	–
CYP2C9*3 (N = 84)	
Gene	
AA (wild type)	84 (100)
AC	–
CC	–
Allele	
A	168 (100)
C	–

*CYP2C9*, *CYP4F2* and *VKORC1* were the genes genotyped. CYP2C9*2 and CYP2C9*3 were not detected. Homozygous CYP2C9*1 or heterozygous CYP2C9*1 were considered putative since we did not genotype for the rest of the other allelic variants

*N* number of study participants, *A* adenine, *G* guanine, *C* cytosine, *T* thymidine

*VKORC1* (A) variant allele was detected to be 12 (6.2%) while those for *VKORC1* (G) was observed at 180 (93.8%) in population studied. Eighty-four patients (87.5%) were homozygous wild-type (G/G) for *VKORC1* while 12 (12.5%) patients were heterozygous (G/A). Homozygous variant (A/A) was not detected in the study population and this is consistent with previous studies which reported that this genotype is very rare (1%) in Africans [29]. This observation however differed from that reported among Asians (55.9%), Caucasians (17.9%), Hispanics (17.8%) and Ashkenazi Jewish (22.7%) populations [27, 28].

Allele frequencies for *CYP4F2* (T) was observed at 96 (41.4%) while that of the wild-type (G) allele was observed at 136 (58.6%). This observation is higher than that reported in African-Americans (11.7%), Asians (30.5%), Caucasians (34.2%), Hispanics (23.3%) and Ashkenazi Jewish (32.8%) populations [17]. For the *CYP4F2*, 28 (24.1%) patients were found to be homozygous wild-type (C/C), 80 (69%) patients were heterozygous for C/T, and 8 (6.9%) patients were homozygous mutant for T/T.

Patients were classified as being on either a high or low depending on their daily warfarin maintenance doses to determine areas where the two populations differed [21]. Eighty-four (59.6%) patients were on low daily warfarin maintenance dose (≤ 5mg/daily) and fifty-seven (40.4%) patients were on high daily warfarin maintenance dose (>5 mg/daily). For female patients, 33 (57%) were on high warfarin dose while 46 (54.8%) were on low maintenance dose. In a multivariate analysis, duration of patients on warfarin therapy and *VKORC1* gene was associated with warfarin dose classification. Duration of patients on warfarin therapy was marginally associated with high warfarin dose (adjusted OR = 1.01 [95% CI 1.00–1.02], p = 0.033) while the odds of heterozygous individuals (G/A) for *VKORC1* gene to have high warfarin dose, compared to individuals with the homozygous (G/G) (adjusted OR = 0.06 [95% CI 0.01–0.63], p = 0.019) compared to individuals with the homozygous (G/G). Age, gender, diagnosis, presence of side effects and other medications were not associated with warfarin dose (p = 0.05) (Table 4).

**Table 4:** Factors associated with high warfarin dosage in patients administered warfarin at Korle-bu Teaching Hospital in Accra

Characteristic	Dosage classification		Crude OR [95% CI]	p value	Adjusted OR [95% CI]^a^	p value
	High	Low				
	N = 57	N = 84				
	n, %	n, %				
Age (years) (median, interquartile range)	46 (33.0–57.0)	48 (36.8–59.0)	0.99 [0.96–1.01]	0.298	0.99 [0.96–1.03]	0.770
Warfarin duration (months) (median, interquartile range)	24 (11–99)	24 (8–61)	1.00 [1.00–1.01]	0.062	1.01 [1.00–1.02]	*0.033*
Gender						
Female	33 (57.9)	46 (54.8)	1.14 [0.58–2.24]	0.713	1.05 [0.40–2.71]	0.928
Male	24 (42.1)	38 (45.2)	1.00			
Body mass index						
Overweight	33 (57.9)	53 (63.1)	0.80 [0.40–1.60]	0.535	–	–
Normal	24 (42.1)	31 (36.9)	1.00			
Diagnosis						
Deep vein thrombosis	21 (936.8)	30 (36.2)	1.00 [0.47–2.11]	0.992	–	–
Atrial fibrillation	2 (3.5)	8 (9.6)	0.36 [0.07–1.81]	0.214	–	–
Pulmonary embolism	8 (14.1)	8 (9.6)	1.42 [0.47–4.28]	0.530	–	–
Mistral valve replacement	26 (45.6)	37 (44.6)	1.00			
Presence of side effects						
Yes	11 (19.3)	16 (19.1)	1.02 [0.43–2.39]	0.970	–	–
No	46 (80.7)	68 (80.9)	1.00			
Other medications given						
Yes	30 (52.6)	46 (54.8)	0.92 [0.47–1.80]	0.803	–	–
No	27 (47.4)	38 (45.2)	1.00			
VKORC1						
GA (heterozygous)	1 (2.6)	11 (19.0)	0.12 [0.01–0.94]	0.043	0.06 [0.01–0.63]	*0.019*
GG (Wild type)	37 (97.4)	47 (81.0)	1.00		1.00	
CYP4F2						
TT (mutant)	5 (9.6)	3 (4.7)	3 [0.59–15.26]	0.186	5.47 [0.61–48.79]	0.128
CT (heterozygous)	37 (71.2)	43 (67.2)	1.55 [0.64–3.77]	0.335	1.91 [0.54–6.80]	0.317
CC (wild type)	10 (19.2)	18 (28.1)	1.00		1.00	

*OR* odds ratio, *CI* confidence interval

^a^Variables with p value <0.02 were entered into the multivariate model in addition to the gene variants VKORC1 and CYP4F2, age, gender and duration on warfarin medication

## Limitations

The study may not have been adequately powered to detect statistically significant differences between patient-specific factors. Allele frequencies of CYP2C9*5, *6, *8, and *11 were not determined in this study due to limitation of resources. Data on patients with unstable warfarin dose and target INRs outside therapeutic range which could have been used as a validation cohort was not collected in this study.

## Conclusion

This study provides data on *VKORC1* and *CYP4F2* variants in an indigenous African population. Duration of patients on warfarin therapy was marginally associated with high warfarin dose. CYP2C9*2 and *3 variants were not detected and may not be the most important genetic factor for warfarin maintenance dose among Ghanaians.

## Abbreviations

INR: international normalized ratio
VKORC1: vitamin K epoxide reductase complex subunit 1
CYP2C9: cytochrome P450 isozyme 2C9
CYP4F2: cytochrome P450 isozyme 4F2
DVT: deep vein thrombosis
AF: atrial fibrillation
HIV: human immunodeficiency virus
AIDS: acquired immune deficiency syndrome
HBV: hepatitis B virus
HCV: hepatitis C virus
BMI: body mass index
KBTH: Korle-bu Teaching Hospital
EDTA: ethylenediamine tetraacetic acid
PCR: polymerase chain reaction
RFLP: restriction fragment length polymorphism
